# Political Institutions and Their Historical Dynamics

**DOI:** 10.1371/journal.pone.0045838

**Published:** 2012-10-03

**Authors:** Mikael Sandberg, Per Lundberg

**Affiliations:** 1 Theoretical Population Ecology and Evolution Group, Department of Biology, Lund University, Lund, Sweden; 2 School of Social and Health Sciences, Halmstad University, Halmstad, Sweden; University College London, United Kingdom

## Abstract

Traditionally, political scientists define political institutions deductively. This approach may prevent from discovery of existing institutions beyond the definitions. Here, a principal component analysis was used for an inductive extraction of dimensions in Polity IV data on the political institutions of all nations in the world the last two centuries. Three dimensions of institutions were revealed: core institutions of democracy, oligarchy, and despotism. We show that, historically and on a world scale, the dominance of the core institutions of despotism has first been replaced by a dominance of the core institutions of oligarchy, which in turn is now being followed by an increasing dominance by the core institutions of democracy. Nations do not take steps from despotic, to oligarchic and then to democratic institutions, however. Rather, nations hosting the core democracy institutions have succeeded in historically avoiding both the core institutions of despotism and those of oligarchy. On the other hand, some nations have not been influenced by any of these dimensions, while new institutional combinations are increasingly influencing others. We show that the extracted institutional dimensions do not correspond to the Polity scores for autocracy, “anocracy” and democracy, suggesting that changes in regime types occur at one level, while institutional dynamics work on another. Political regime types in that sense seem “canalized”, i.e., underlying institutional architectures can and do vary, but to a considerable extent independently of regime types and their transitions. The inductive approach adds to the deductive regime type studies in that it produces results in line with modern studies of cultural evolution and memetic institutionalism in which institutions are the units of observation, not the nations that acts as host for them.

## Introduction

Traditionally, political institutions are defined deductively in political science. Already Aristotle deductively distinguished between democracy, oligarchy and tyranny on the basis of his taxonomy of constitutions [1]. Such distinctions are still common. For example, the major institutions of democracy, ‘parliamentarism’ and ‘presidentialism’, are defined in term of who appoints the government according to the constitution. Similarly, ‘democracy’, in modern political theory, has been defined as “that institutional arrangement for arriving at political decisions in which individuals acquire the power to decide by means of a competitive struggle for the people's vote” [2], or a political system “responsive to all citizens” [3]. These definitions are not results of prior empirical tests, experiments or investigation of all observed institutions, but are classifications developed on the basis of common knowledge, logic and conceptual distinctions. One problem with this conceptually rather than empirically driven approach is that one can never be sure that these man-made classifications of institutions—or other conceptually defined phenomena—correspond to actually existing conditions. A serious consequence of this approach is that institutions that have not been conceptually defined cannot be detected. If natural sciences had followed the research principles of political science, no discoveries beyond *a priori* conceptualization could have been made.

One can argue that institutions, just like genes, are particulate units. An institution, just like a gene, exists or not. Further, one could argue that the possible combinations of non-existing and existing institutions at any time and social space are beyond quantitative limits; any political or social unit may have an infinite number of combinations of institutions and the lack of them. In analogy with Dawkins' expression, “however many ways there may be of being alive, it is certain that there are vastly more ways of being dead, or rather not alive” [4], one task for the political or social scientist should be to detect exactly what combinations of existing and non-existing institutions there actually are in each political system over time. For example, rule of law, or the lack of rule of law, can be combined with parliamentarism or the lack of parliamentarism (such as presidentialism), in turn combined with various types of elections systems. However, considering the number of institutions, nation-states and years of their existence, this task is a challenge for an industry of political scientist. The idea in this study is therefore to limit the endeavor to the investigation of already existing institutional data for an inductive exploration and analysis. What key dimensions among institutional variants at nation-state level can be found using already existing data? How have these institutional dimensions evolved over time? What nation-states have these institutions been able to “invade” and for what periods in time? What can we learn from this exploration of institutional data inductively in relation to traditional deductive approaches? In particular, how do these inductively extracted institutions relate to the regime types understood and defined deductively?

In the definitions of ‘institutions’, as well as in the approaches to studying them, political science has been greatly influenced by institutional economics (such as [5], [6]–[8], for an overview see [9]). In particular, North's understanding of institutions as “rules of the game” or more specifically the “humanly devised constraints that structure political, economic and social interaction” [6] has been hugely influential. Ostrom has applied a similar concept of institutions in various studies of the commons (exploitation of common local resources) in ways that come closer to a political economics of political science [10]. Traditional subfields of political science, however, have made historically fewer contributions to institutional theory (see however [11], [12]–[14]), despite the ancient traditions from Aristotle. Instead, deductively motivated empirical analyses of institutions are more common in political science; in particular studies of what is first defined as democracy and then explained in terms of ‘requisites’, determinants and diffusion patterns ([15]–[45] to name of few studies in this field). Interestingly, compared with the numerous studies of democracy and its correlates, much less research has focused on non-democratic regime-types. This is unfortunate, since the potential for democracy's survival depends on the regime-type it succeeds. In particular Dahl [4] and Linz and Stepan [28] acknowledge this understanding of historical dynamics in their analysis of fundamental pathways toward a consolidated democracy.

## Materials and Methods

If institutional economics is richer in theory, institutional political science is richer in data. In particular, the Polity IV data set is interesting since it covers all nation-states with a population of more than 500,000 inhabitants for all years starting from 1800 [27], [39], [46]–[49]. The Polity IV data set includes a number of institutional (ordinal scale) variables. These variables have typically been used for measuring the degree of autocracy and democracy as well as the institutional steps between the two (“anocracy”). As described in their Polity IV codebook [47], the autocracy value subtracted from the democracy value gives the “Polity score” from -10 (strongly autocratic) to 10 (strongly democratic). The variables are the product of the conceptual work of Eckstein and Gurr [49] and though it is thus a list of institutions defined deductively, the resulting variable list of institutions is the result of pragmatic operationalization (see especially [47], p. 16). Polity IV is the most impressive data set of political institutions from an explorative point of view. The rational in this study can be formulated as “it is more likely you find the key the more lampposts there are”, and it can be economical to search there before investing in further illumination.

The institutions that the Polity IV data set measures are, first, three variables of executive recruitment: (1) regulation of chief executive recruitment, (2) competitiveness of executive recruitment, and (3) openness of executive recruitment; second, one variable on the independence of executive authority: (4) executive constraints (decision rules); and, third, two variables on political competition and opposition: (5) regulation of participation, and (6) competitiveness of participation. Since these six variables are ordinal scales, each of their values can be transformed into separate variables. In this case, we end up with 30 institutions with values 0 if they do not occur in a country a particular year, or 1 if they do (see Appendix S1). These 30 variables of political institutions are strongly correlated and it is obviously the case the there is a significant redundancy among them. In addition, they suffer from group-wise singularity, since they are created from original variables in which the values are mutually exclusive. What we need is therefore a technique to find the most important, uncorrelated dimensions behind the values of these variables, i.e. to reduce the number of variables into key dimensions and at the same time splitting up their group-wise singularities. This is why a principal component analysis (PCA) with strict selection criteria is used. PCA is a procedure for reducing the number of variables in both cross section and time series data in cases where no causation is assumed between the variables. PCA reduces the number of variables into a set of principal components that are uncorrelated and account for most of the variance. Each principal component is a linear combination of the original variables so it is often possible to ascribe meaning to what the components represent, in this case an “institutional dimension” (correlated institutions that differ from other dimensions of correlated institutions). PCA is similar to factor analysis, but has the advantage that it does not rely, as opposed to factor analysis, on any assumption of underlying causal structure [50], [51]. In this case the analysis has been based on the Polity IV original data set, covering the years 1800–2007. The procedure has been the following.

Polity data was first transformed into the 30 dummy variables as described above and in the Appendix S1. The 30 variables were included in an initial principal component analysis, using varimax (orthogonal) rotation on a covariance matrix. Applying a number of selection criteria in repeated PCAs, the number of valid variables was reduced for arriving at a robust solution and avoiding singularity. Variable finally selected were only those that: (1) gave a solution with commonalities ≥0.50, (2) high loading (≥0.50) on only one component, (3) made possible the reproduction of acceptable levels of commonalities and the same components in repeated analyses with randomly selected halves of the sample, (4) had values ≥0.50 in the diagonal table of anti-image correlations, (5) had a value ≥0.50 on the Kaiser-Meyer-Olkin measure of sampling adequacy, (6) had a significant value (<.000) on the Bartlett test of sphericity, and (7) a value ≥0.70 on Chronbach's alpha for the extracted variables of each principle factor (alpha = 0.716, 0.878 and 0.681 for the three dimensions, indicating a somewhat weaker reliability of the third component, based on the two core institutions of despotism, should they be used as a scale, something which is not the case in this study). Only seven of the 30 original variables passed these tests. In three cases were two values from the original variables kept, thus indicating that the problem of singularity had been avoided to a great extent. In addition, component loadings found to be ≥3.0 were excluded as outliers (642 of 15520, or 4,2%), as recommended by the SPSS documentation.

## Results

The principal component analysis on Polity IV data 1800–2007 reveal three components which together explain more than 83 percent of the total variance of the seven variables that passed the tests listed above (table 1).

**Table 1:** Principal Component Analysis of Polity IV institutional variables.

	Components		
	1	2	3
1. Regulation of Participation: regulated	.921		
2. Competitiveness of Participation: competitive	.916		
3. Executive Constraints: parity or subordination	.820		
4. Competitiveness of Participation: factional		.940	
5. Regulation of Participation: sectarian		.925	
6. Executive Constraints: unlimited authority			.900
7. Openness of Executive Recruitment: closed			.802

Note: Principal component analysis of a covariance matrix, using the varimax reduction method. Selection criteria: see text.

### The first dimension: core institutions of democracy

In the first dimension we find the following institutions (values on Polity IV variables): (1) Regulation of Participation: regulated, (2) Competitiveness of Participation: competitive, and (3) Executive Constraints: parity or subordination.

Regulated and competitive participation and subordination of the executive is at heart of democracy. The three institutions are part of an institutional dimension to which democracies belong. Regulated participation indicates that there are binding rules for when and how political preferences are expressed. Both one-party states and Western democracies exhibit regulated participation but they do so in different ways. Marshall and Jaggers state that ”regulated” implies that [47]:

relatively stable and enduring political group regularly compete for political influence and positions with little use of coercion. No significant groups, issues, or types of conventional political action are regularly excluded from the political process.

The next variable in the analysis supports this conclusion. Second highest ranking on this first dimension is given competitiveness of participation. Marshall and Jaggers state that this value indicates “stable and enduring, secular political groups which regularly compete for political influence at the national level” [47]. Under the third institution parity and subordination in executive constraints, accountability groups “have effective authority equal to or greater than the executive in most areas of activity”, such as “a legislature, ruling party, or council of nobles initiates much or most important legislation” (p. 25).

Nations with all three core institutions of democracy may or may not be democratic as defined by the Polity score, for which 6 is considered the threshold value for being democracy, as recommended on the Polity IV home page. Several countries are defined as democracies in Polity score terms without having acquired the three core democracy institutions, such as Albania, democratic since 2002, Argentina, democratic in 1973–75 and since 1983, and Armenia, democratic in 1991–94. In fact there are 118 countries that have at least one year been democratic, while only 43 countries have ever fulfilled the criteria of having all the three institutions of the core democracy dimension. On the other hand, the 43 countries with the core democracy institutions in place are not always considered democratic in terms of the Polity score the same years. The reason is that the Polity score is the sum of both institutionalized democracy and (the negative value of) institutionalized autocracy. A value over 6 can therefore be based on a very high value of democracy and a low (negative) value of autocracy.

**Figure 1:**
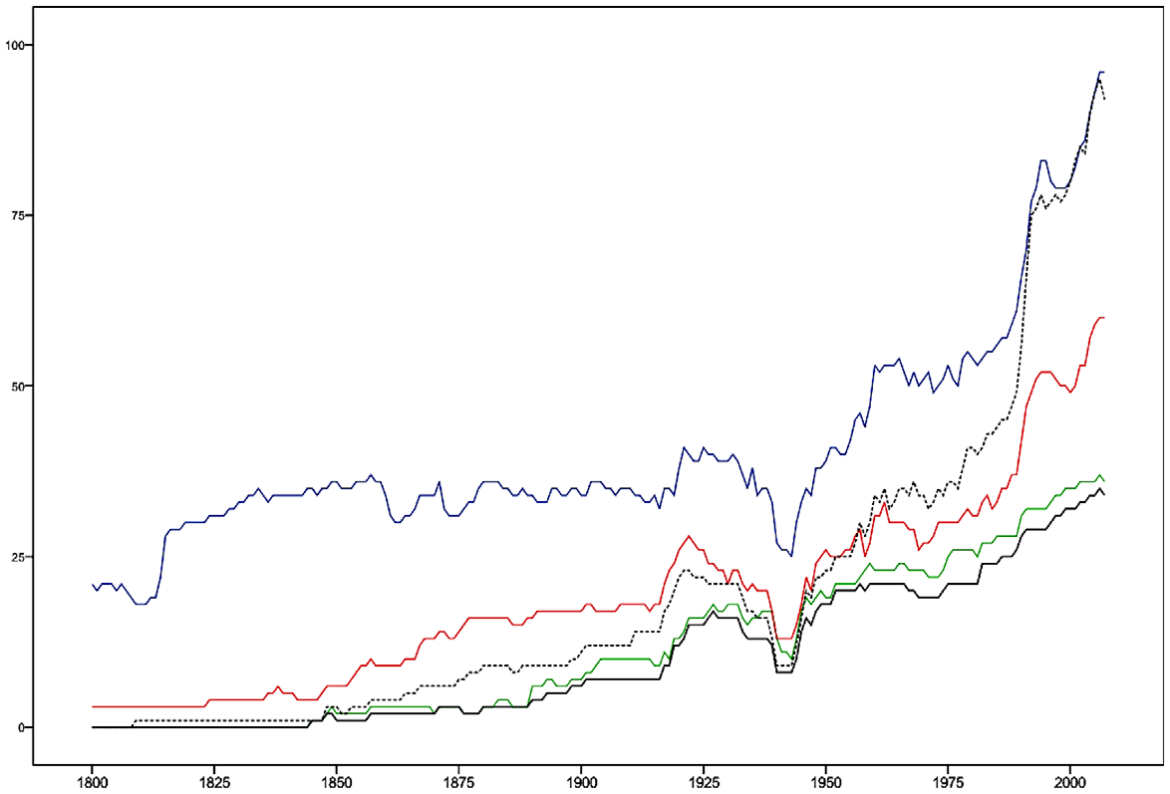
Historical dynamics of the democracy dimension. The figure 1 shows the number of nations having the three core institutions of democracy; Regulation of executive recruitment: regulated (blue); Competitiveness of participation: competitive (green); Executive constraints: parity of subordination (red). Nations may have none, one, two or three of these institutions. The sum of those nations that have all three of them is indicated (black). In addition, the number of nations being democracies in the sense of having 6 or more on the Polity score (as revised by the Polity IV project) is given (dashed).

In some cases, the Polity score definition of democracy and acquisition of the three core institutions of democracy coincide. Australia, for example, has the three core democratic institutions from 1901- and indeed more than 6 on the Polity score from the same year. Austria, on the other hand, had the core democracy institutions from 1946-, while being democratic in the sense of having more than 6 on the Polity score already in 1920–32. Belgium had the three core institutions in 1919–38 and in 1944–2006, while its Polity score is above six already from 1843. Canada had the core democracy institutions from 1921, but was democracy already from 1888. Chile had gained the three core democratic institutions from 2006, but was considered democratic in Polity score terms between 1964 and 1972, and from 1989. As Chile's case illustrates, a society can be considered democracy without having all critical democratic institutions in place, and one might hypothesize that the nations lacking the core democratic institutions are more likely to become victims of reversals into non-democratic regime types than those that have them. On the other hand, democracy may also lead to the subsequent introduction of the lacking core democratic institutions that may consolidate democracy. In fact, in all cases, except Egypt 1922–27, nations are considered democratic in Polity score terms the same year or prior to gaining the three core democracy institutions. Democracy as regime type obviously may “breed” or innovate its own consolidating institutions, if they are not at hand initially (see Appendix S2). Figure 1 describes the historical dynamics of this dimension in terms of how many nations acquired each of the three core institutions of democracy.

If we instead look at the historic evolution of the core democracy dimension of institutions, the US as first polity gains the core three institutions in 1845, but in 1849–1871, in connection with the Civil War, fails to fulfill these criteria. Switzerland from 1848 is the only country with an unbroken sequence of years with the required values on the three dimension variables until the present day. In 1857, as third nation acquiring the core democracy variable values, we find New Zealand. In 1880, Greece, as fourth country, meets the criteria. In 1890, Costa Rica becomes the fifth polity of the democracy dimension, and in 1898 Norway becomes the sixth. By 1927, we have 17 nations of that dimension: Australia, Belgium, Canada, Costa Rica, Denmark, Egypt, Estonia, Finland, Greece, Ireland, the Netherlands, New Zealand, Norway, Sweden, Switzerland, the United Kingdom, and the United States. In connection with the World War II, the number decreased to eight. From the 1950's, with the exception for some of the years in the late 1960s and the early 1970s, the increase of nations with the three institutions of the democracy dimension is monotonic. In 2006, the number of nations having the three core democracy institutions is 35 (figure 1).

### The second dimension: oligarchy

The second component given by the principal component analysis in table 1 is called ‘oligarchic’ due to the factional or sectarian institutions involved: Competitiveness of Participation: factional and Regulation of Participation: sectarian. The variables of the factional or sectarian institutions focus on participation and regulation of participation. The factional aspect of competition in participation is defined by Marshall and Jaggers as polities “with parochial or ethnic-based political factions that regularly compete for political influence in order to promote particularist agendas and favor group members to the detriment of common, secular, or cross-cutting agendas” [47]. Factional competitiveness is thus typical to divided societies that have not created sufficient institutions for democracy. The second variable, regulation of participation: sectarian, imply that political demands are characterized by “incompatible interests and intransigent posturing among multiple identity groups and oscillate more or less regularly between intense factionalism and government favoritism” [47]. The term ‘oligarchic’ in this case implies the institutions where one identity group favors group members in central allocations and restrict competing group's activities and when significant portions of the population historically have been excluded from access to power.

**Figure 2:**
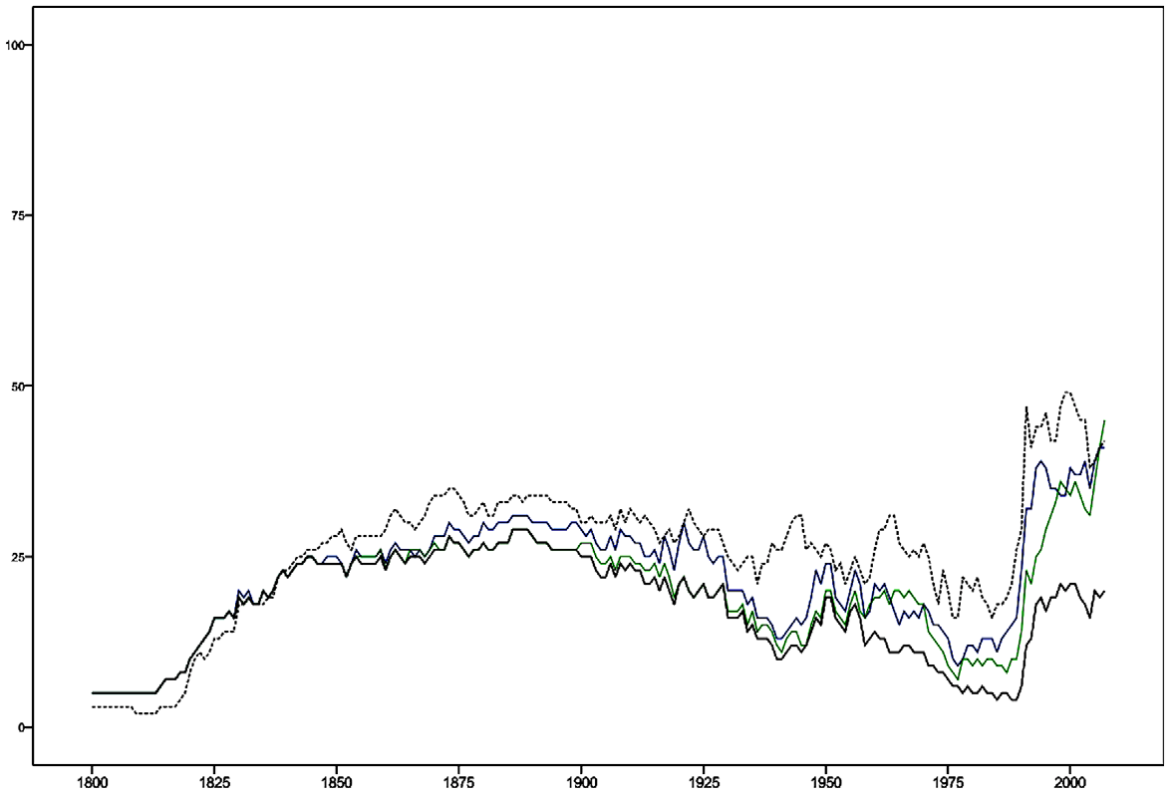
Historical Dynamics of the Factional/Sectarian (Oligarchy) Dimension. The figure 2 shows the number of nations having the two core institutions of oligarchy; Competitiveness of participation: factional (blue); Regulation of participation: sectarian (green). Nations may have none, one, or two of these institutions. The sum of those nations that have both of them is indicated (black). In addition, the number or nations being “anocracies” in the sense of having −5 to 5 on the (revised) Polity score is given (dashed).

In 2000, Algeria, Angola, Burundi, Chad, Estonia, Ethiopia, Guinea, Iran, Ivory Coast, Kazakhstan, Liberia, Malaysia, Sri Lanka, Tajikistan, Togo, Tunisia, Turkey, Yemen, and Zimbabwe (19 nations) had positive values on the two variables in the faction/sectarian (oligarchic) dimension. The dimension's all time high was in 1886–1889, when 29 countries had both core oligarchy institutions. Historical dynamics are described in Figure 2.

### The third dimension: despotism

In the third principal component variable we find the variable values Executive Constraints: unlimited authority and Openness of Executive Recruitment: closed.

An inspection of the two variables of this third dimension of principal component reveals that we deal here with societies in which there “are no regular limitations on the executive's actions”, such as [47]:

1. Constitutional restrictions on executive action are ignored.
2. Constitution is frequently revised or suspended at the executive's initiative.
3. There is no legislative assembly, or there is one but it is called and dismissed at the executive's pleasure.
4. The executive appoints a majority of members of any accountability group and can remove them at will.
5. The legislature cannot initiate legislation or veto or suspend acts of the executive.
6. Rule by decree is repeatedly used.

Such regimes are strongly correlated with polities in which executive recruitment is closed [47]:

1. Chief executives are determined by hereditary succession, e.g. kings, emperors, beys, emirs, etc. who assume executive powers by right of descent.
2. Executive recruitment is closed to everyone who is not a member of the hegemonic party/faction that controls the government.

**Figure 3:**
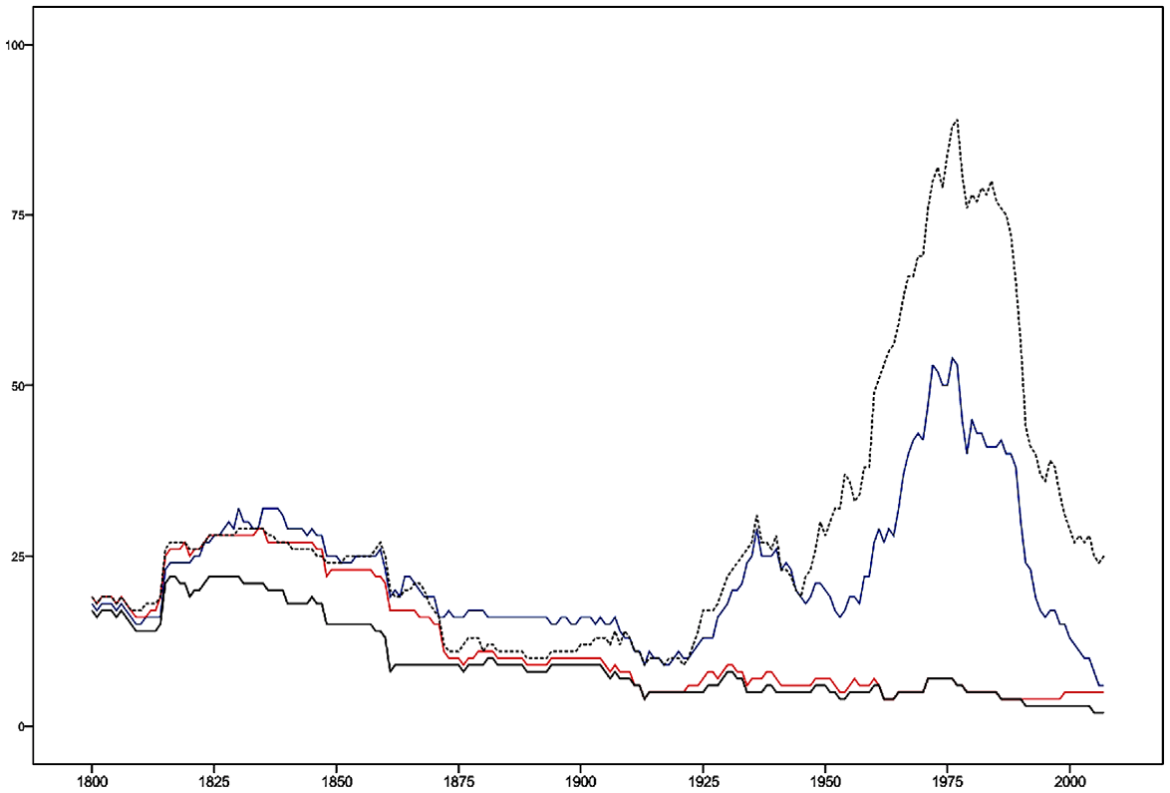
Historical dynamics of the despotism dimension. The figure 3 shows the number of nations having the two core institutions of despotism; Executive constraints: unlimited authority (blue); Openness of executive recruitment: closed (red). Nations may have none, one, or two of these institutions. The sum of those nations that have both of them is indicated (black). In addition, the number of nations being “autocracies” in the sense of having −6 or less on the (revised) Polity score is given (dashed).

For these reasons it is natural to call the dimension ‘despotism’ in modern terminology, corresponding to the otherwise tempting Aristotelian term ‘tyranny’. Nations exhibiting the positive values on the variables of this dimension at least one year since the mid 1990s are only three: Bhutan, Qatar and Saudi Arabia. In the periods 1816–17 and 1824–1830, however, there were 22 nations with the two core despotic institutions. Historical dynamics of this dimension is given in Figure 3.

### Outliers

As mentioned, there were 642 outliers in the material, constituting the 4.2 percent of the 15520 country-year cases with higher loading than the 3.0 recommended as ceiling by SPSS documentation for the saved component loadings. Typically, these countries did not combine despotic unlimited authority in executive recruitment with the expected closed executive recruitment of the despotic dimension. Since the late 1920s, a substantial number of these political systems instead combined despotic unlimited authority in executive recruitment with a factional regulation of participation. They thus exhibited other combinations of institutions than those of the major dimensions.

### Comparative dynamics of institutional dimensions

**Figure 4:**
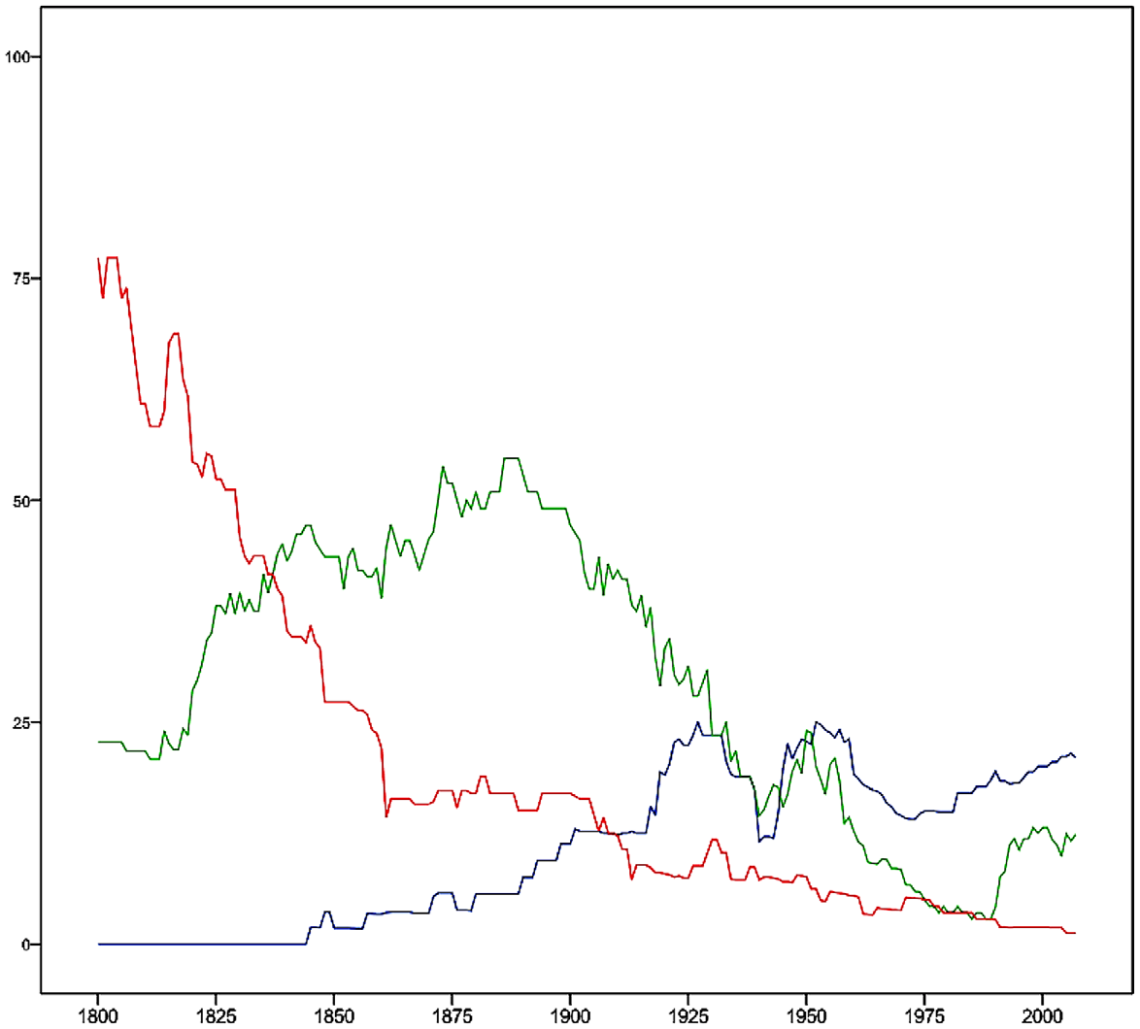
The three institutional dimensions the last two centuries. The figure 4 shows how many percent of the nations in the world that had all the core institutions of each of the three dimensions described in the figures 1, 2, 3 above. The world system of nation-states is first dominated by the despotism dimension (red), then by the oligarchy dimension (green), and lastly by the democracy dimension of institutions (blue). Their subsequently declining dominance suggests a growing diversity in institutional set ups of nations.

The three institutional dimensions have interesting historical dynamics, as is obvious from the figure 4, in which percentages of country cases with all the core institutions of despotism, oligarchy and democracy are given. In the last two centuries, the despotism dimension decreases from over 70 percent of all nations in 1800 to less than 5 percent in 2000.

The oligarchic dimension increases in the mid 1800s, with a peak around 1885, and then declines to around five percent in the 1980s, with two recoils around 1950 and 2000. The democracy dimension is slowly increasing with wave-like steps ahead in the beginning of the last century, in the early twenties, thirties and fifties, but recoiling into a rift around the Second World War. From the three dimensions one can hypothesize a substitution in the sense that oligarchic institutions succeed the despotic, and the democracy dimension replaces the oligarchic. This hypothesis does not find support in evidence at nation-state level, however (see Table 2 and Appendix S2). On the contrary, the nations having the core three democratic institutions tend previously to have had neither the core despotic nor the core oligarchic institutions. For instance, only Austria, Belgium, Chile, France, Germany, Hungary, Italy, Japan, the Netherlands, Portugal, Spain and Uruguay (12 nations) have both had oligarchic and democratic core institutions, while 22 nations have had the three core democratic but never the core oligarchic institutions. Only eight of the 43 nations that ever have gained the core democratic institutions have also previously had core despotic institutions; Austria, Denmark, Egypt, Japan, the Netherlands, Portugal, Spain, and Sweden. These facts suggest a path-dependency among political institutions in the sense that an oligarchic or despotic past seems to be an impediment to later acquisition of the core democracy institutions. Indeed, an OLS regression of the sum of values of the core despotism and oligarchy institutions on the sum of values on the democratic dimension explains only 0.076 of the variance (R squared), but the standardized regression coefficient of the oligarchy dimension is −0.21*** as compared with −0.18*** for the despotism dimension, indicating that oligarchy has slightly more negative influence than despotism on acquisitions of the core democracy institutions.

**Table 2:** The number and frequencies of country-year institutional combinations of the three institutional dimensions.

			Number of the three core democracy institutions								Total	
			None		One		Two		Three			
			n	%	n	%	n	%	n	%	n	%
None of the two core despotism institutions	Number of the two core oligarchy institutions	None	2520	37.6	895	13.4	1177	17.6	2105	31.4	6697	100
		One	490	38.2	439	34.2	353	27.7	0	0	1282	100
		Two	1246	54.3	841	36.7	206	9.0	0	0	1293	100
	Total		4256	41.4	2175	21.2	1736	16.9	2105	20.5	10272	100
One of the two core despotism institutions	Number of the two core oligarchy institutions	None	1864	78.7	497	21.0	0	0	6	.3	2367	100
		One	73	81.1	17	18.9	0	0	0	0	90	100
		Two	608	77.4	50	6.4	128	16.3	0	0	786	100
	Total		2545	78.5	564	17.4	128	3.9	6	.2	3243	100
Two of the two core despotism institutions	Number of the two core oligarchy institutions	None	0	0	1456	100	0	0	0	0	1456	100
		One	0	0	10	100	0	0	0	0	10	100
		Two	0	0	539	100	0	0	0	0	539	100
	Total		0	0	2005	100	0	0	0	0	2005	100
Total	Number of the two core oligarchy institutions	None	4384	41.7	2448	27.1	1177	11.2	2111	20.1	10520	100
		One	563	40.7	466	33.7	353	25.5	0	0	1382	100
		Two	1854	51.2	1430	39.5	334	9.2	0	0	3618	100
	Total		6801	43.8	4744	30.6	1864	12.0	2111	13.6	15520	100

Interestingly, the “market saturation” is decreasing, that is, a much lower share of the world's nations are exhibiting any of the dimensions in the 20^th^ than in the 19^th^ century. In the 1800s, more than three fourths of the nations belonged to the despotism dimension, and nearly the rest the oligarchic. As the oligarchic dimension expands in the world system, at the cost of despotism, some countries are lost and not yet influenced by the core democracy institutions. The tide is lowest during World War II: together the despotism, oligarchy and democracy dimension influence less than half of the nations. After the war, the core democracy institutions gain momentum and around the end of the 1900^th^ century, its “market share” is more than 20 percent. Together with oligarchic and despotism institutions, around a third of the nation-states are influenced. The rest of the 160 nations have none of these dimensions in full. So, as democratic institutions substitute the oligarchic and the despotism dimensions, there is an increasing share of country cases with idiosyncratic institutional set ups, indicating an increasing institutional diversity. Not only are there many countries that are not influenced by any dimension in full. Some nations are instead affected by more than one dimension and other institutions than those included in the three major dimensions. The situation is quantified in table 2 and described in country case detail in Appendix S2.

As we can notice in table 2, there are 539 country and year cases with both the despotism and oligarchy dimensions present. In addition, they all have one of the three institutions of the democratic dimension. Even among countries with a maximum value on the democracy dimension, there are 6 country and year cases (Egypt 1922–1927) with one of the despotism institutions. So, nation-states may host several institutional dimensions depending on the exact composition of constituent institutions.

### Comparing deductive and inductive definitions

**Figure 5:**
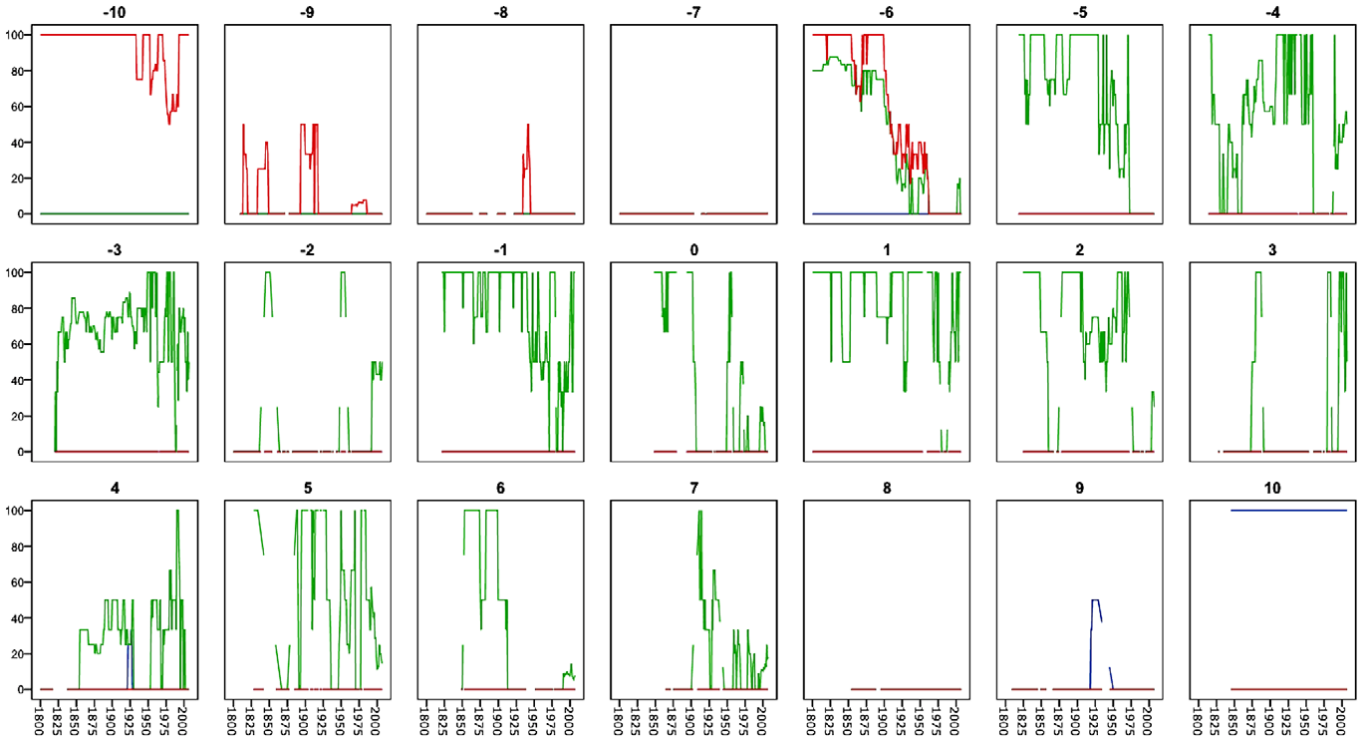
Institutional dimensions and Polity scores. The figure 5 divides figure 4 depiction of how many percent of the nations in the world had all the core institutions of each of the three dimensions described in figures 1, 2, 3 into the values nations have on the Polity score from strongly autocratic (−10) to strongly democratic (10). The democracy dimension dominates among the Polity score 10 nations, reveal a short wave in Polity score 9 nations around 1925, but is otherwise absent. The despotism dimension dominates in Polity score −10, exhibits increases in various periods in Polity score −9 nations in the early 1900s and the first half of the 19^th^ century. The oligarchic institutional dimension is scattered among nations with a Polity score −6 to 7. Nations having the three core democracy institutions (blue); Nations having the two core factional/sectarian (oligarchy) institutions (green); Nations having the two core despotism institutions (red).

One should keep in mind that the evolution of the institutional dimensions occurs across nations with varying degree of democracy and autocracy as defined by the Polity score [47]. In figure 5, we see that institutions from the democracy dimension dominate only in the Polity score 10 value (strong democracy), while the despotism dimension is found in the −10 score (strong autocracy) and occasionally in the −10 to −6 scores. The oligarchy dimension is frequent in the −6 to 7 revised Polity score, but not systematically, indicating a weak correlation between these dimensions and the deductively constructed Polity score (Pearson's r: 0.721** with the democracy dimension, −0.016 with the oligarchy dimension and −0.617** with the despotism dimension). Only the inductively extracted core institutional democracy and despotism dimensions are strongly related to the revised Polity score, which in turn is created from two Polity IV variables—the autocracy value subtracted from the democracy value. Normally, as described on the Polity IV home page, countries with a Polity score +6 to +10 are deductively defined “democratic”. Results here show that this deductive regime classification does not correspond to inductively extracted core institutional dimensions. In particular, the deductive regime type classification is void of oligarchy as regime type, despite the fact that, at institutional level, there is one core oligarchic institutional dimension extractable in the material.

The figure 5 clearly shows how deductive versus inductive approaches to institutional analysis produce quite different results. Clearly, the deductive and inductive approaches to classification produce answers to different questions. The deductive approach answers the question to what degree the nations' regime types fulfill the requirements we define as democracy versus autocracy (or “anocracy”, as suggested by Polity IV). But the core democracy, oligarchy and despotism dimensions do not fit neatly into the deductive autocracy-anocracy-democracy scale of the Polity score. In particular, the core oligarchy dimension is scattered in several Polity score values and years. The deductive scale and the inductive dimension obviously cut data quite differently. The inductive approach instead produces information about major dynamics historically at institutional level. For instance, we are informed that nations may simultaneously be hosts of core institutions of different political dimensions. The two approaches thus produce knowledge from the regime versus the institutional perspectives. The deductive approach reveals historical degree of nation-state democracy as we conceptualize it in one case. The inductive approach gives insights into dimension of political institutions as they evolve over time across all regime types in all nations. This fact implies that the results of this study do not necessarily contradict results of recent studies of dynamics in transitions between autocracy and democracy [43], but that units, levels and perspectives of analysis are different. The inductively found variable values of the three institutional dimensions open up for a new approach to studies of the underlying institutional dynamics of the political regimes as we know them. Rather than only considering each nation each year a democracy, “anocracy” or autocracy with a specific Polity score and specific institutions at hand, we discover that institutions exhibit dynamics below and in interaction with changes at the regime type level. In some cases, core institutions of more than one regime-type invade regimes; in others there is a lack of any of the core institutions in a regime. We therefore conclude that the political meso-scale (the political institutional level) and the macro-scale (regime types) partly have decoupled dynamics and that the derivation of regime type from institutions is not always straightforward. The increase of the democratic regime type over time does imply that a certain set of institutions also becomes more widespread, but not unambiguously so. A smaller fraction of the world's polities cannot easily be categorized as any given regime type. The reason for this increase in institutional diversity calls for an evolutionary understanding of how the meso- and macros-scales of political culture are interacting.

## Discussion

Traditionally, political scientists define democracy as well as other political institutions conceptually and deductively. Attempts are made to fit polities and nations into pre-defined categories and classes. This approach may prevent from discovery of actually existing institutions or dimensions of institution that are outside of the scope of the definitions. Here, a principal component analysis was used as a tool for inductive extraction of institutional dimensions in the Polity IV data. Three dimensions were revealed, based on seven institutional variables that passed a strict series of test for a principal component analysis. The three recovered dimensions correspond to intuitively sensible categories, but now being the result of posterior classification. The overall time dynamics of those dimensions are the core institutions of the regime types democracy, oligarchy and despotism. The despotism dimension was historically succeeded by the oligarchic dimension, which in turn was followed by the democratic. Curiously, this result is congruent with Aristotle's classic distinctions between the three ‘deviant’ (unjust) constitutions of city-states: democracy, oligarchy and ‘tyranny’ (!). We also show that some country cases are influenced by none of these, some only by one dimension, while a few have had institutions from two or three of these core institutional variables. Nation-states' regimes do not typically take steps from core institutions of despotism, to oligarchy, and finally to democracy, however. In fact, core institutions of despotism and oligarchy of a regime impede the later acquisition of core democratic institutions, even if there are a number of exceptions to this rule. Our conclusion is therefore that, instead of only considering each nation each year a democracy or autocracy with a specific Polity score, we should also study how institutional dimensions and institutions evolve historically across nations' regime types. Our results are in line with modern studies of cultural evolution and evolutionary (memetic) institutionalism [43], [54]–[56], where institutions are considered the units of observation, not the nations or polities that act as host for them. Results here add to these new areas of research in suggesting that the relationship between the meso-scale dimensions of political institutions and the macro-scale regime types may be considered analogous to the biological distinction between genotypes and phenotypes. In biology, many quantitative traits are genetically canalized [52], [53], i.e., insensitive to the exact genetic architecture underlying the trait development. Small to moderate genetic changes are invisible at the trait level. In the same vein, political regime types can apparently also be canalized; the underlying institutional architecture can vary; sometime quite considerably, yet the regime type prevails. To understand the interactions between the dynamics of the political institutions and the transitions of regime-types is a critical challenge for a modern, evolutionary political science.

## Supporting Information

Appendix S1 **The 30 institutional dummy variables extracted from the Polity IV data set.**
(DOC)

Appendix S2 **List of nation-states and year of core despotism, oligarchic and democracy institutions, and polity score “democracy”.**
(DOC)
