# Supplementary material for: Political Institutions and Their Historical Dynamics
Source: PLoS One. 2012 Oct 3;7(10):e45838. doi: 10.1371/journal.pone.0045838 (PMC3463615; doi:10.1371/journal.pone.0045838)
Supplement: Appendix S1 — The 30 institutional dummy variables extracted from the Polity IV data set. (DOC) [file pone.0045838.s001.doc]

Supporting Information file 1. The 30 institutional dummy variables extracted from the Polity IV data set

| 1 | Regulation of Executive Recruitment: unregulated | 16 | Executive Constraints: intermediate 3/5 |
| --- | --- | --- | --- |
| 2 | Regulation of Executive Recruitment: designational/transitional | 17 | Executive Constraints: substantial |
| 3 | Regulation of Executive Recruitment: regulated | 18 | Executive Constraints: intermediate 5/7 |
| 4 | Competitiveness of Executive Recruitment: unregulated | 19 | Executive Constraints: parity or subordination |
| 5 | Competitiveness of Executive Recruitment: selection | 20 | Regulation of Participation: unregulated |
| 6 | Competitiveness of Executive Recruitment: dual/transitional | 21 | Regulation of Participation: multiple identity |
| 7 | Competitiveness of Executive Recruitment: election | 22 | Regulation of Participation: sectarian |
| 8 | Openness of Executive Recruitment: unregulated | 23 | Regulation of Participation: restricted |
| 9 | Openness of Executive Recruitment: closed | 24 | Regulation of Participation: regulated |
| 10 | Openness of Executive Recruitment: dual designation | 25 | Competitiveness of Participation: not applicable |
| 11 | Openness of Executive Recruitment: dual election | 26 | Competitiveness of Participation: repressed |
| 12 | Openness of Executive Recruitment: open | 27 | Competitiveness of Participation: suppressed |
| 13 | Executive Constraints: unlimited authority | 28 | Competitiveness of Participation: factional |
| 14 | Executive Constraints: intermediate 1/3 | 29 | Competitiveness of Participation: transitional |
| 15 | Executive Constraints: slight to moderate | 30 | Competitiveness of Participation: competitive |
