# Supplementary material for: Political Institutions and Their Historical Dynamics
Source: PLoS One. 2012 Oct 3;7(10):e45838. doi: 10.1371/journal.pone.0045838 (PMC3463615; doi:10.1371/journal.pone.0045838)
Supplement: Appendix S2 — List of nation-states and year of core despotism, oligarchic and democracy institutions, and polity score “democracy”. (DOC) [file pone.0045838.s002.doc]

Supporting Information file 2. List of nation-states and year of core despotism, oligarchic and democracy institutions, and polity score ”democracy”

| *Two core despotism institutions* | *Two core oligarchy institutions* | *”Democracy” as value 6 or more on the Polity score* | *Three core democracy institutions* |
| --- | --- | --- | --- |
| Afghanistan 1800-1952 | Afghanistan 1800-1934 |  |  |
|  |  | Albania 2002- |  |
|  | Argentina 1825-28,  1835-1929 | Argentina 1973-75,  1983- |  |
|  |  | Armenia 1991-94 |  |
|  |  | Australia 1901- | Australia 1901- |
| Austria 1800-1804, 1806-60 | Austria 1848-1917 | Austria 1946- | Austria 1946- |
|  |  | Bangladesh 1971-72,  1991-2006 |  |
|  |  | Belarus 1991-1994 |  |
|  | Belgium 1853-1912 | Belgium 1853-1913,  1915-38,  1944- | Belgium 1919-38,  1944-2006 |
|  |  | Benin 1991- |  |
| Bhutan 1907-2004 |  |  |  |
|  | Bolivia 1825-63, 1873-75, 1880- | Bolivia 1982- |  |
|  |  | Botswana 1966- |  |
| Brazil 1824-88 | Brazil 1824-1929, 1947- | Brazil 1946,  1958-60,  1985- |  |
| Bulgaria 1881-82, 1894-1912, 1914-17 | Bulgaria 1886-93, 1918-33 | Bulgaria 1990- |  |
|  |  | Burundi 2005- |  |
|  |  | Canada 1888- | Canada 1921- |
|  | Chile 1880-90 | Chile 1964-1972,  1989- | Chile  2006- |
| China 1800-59, 1862-1910 | China 1800-59, 1862-1936, 1946-48 |  |  |
|  | Gran Colombia 1821-30  Colombia 1832-59,  1861-66, 1886-1929, 1948- | Colombia 1867-1885,  1957- |  |
|  |  | Comoros 2004- |  |
|  | United Province CA 1824-1838 | Costa Rica 1875- | Costa Rica 1890- |
|  |  |  |  |
|  |  | Cyprus 1960-61,  1968- | Cyprus  1974- |
|  |  | Czech Republic 1993- | Czech Republic 1993-2005 |
|  |  | Czechoslovakia 1918-1938,  1990-92 | Czechoslovakia  1945-46 |
| Denmark 1800-48 |  | Denmark 1911-1939,  1945- | Denmark 1915-39,  1945- |
|  | Dominican Rep 1844-60, 1865-1913,  1925-29 | Dominican Rep 1978- |  |
|  |  | East Timor 2002 |  |
|  | Gran Colombia 1821-30  Ecuador 1830- | Ecuador 1979-2006 |  |
| Egypt 1930-33 | Egypt 1930-33, 1935- |  | Egypt 1922-27 |
|  | United Province CA 1824-1838  El Salvador 1841-54, 1858- | El Salvador 1984- |  |
|  |  | Estonia 1917,  1919-1933,  1990- | Estonia  1919-32 |
|  | Ethiopia 1855-1929 |  |  |
|  |  | Fiji 1970-86,  1999,  2004-05 |  |
|  |  | Finland 1917-1930,  1944- | Finland 1919-29,  1944- |
|  | France 1814-47 | France 1876-1939,  1946-1957,  1969- | France 1930-39,  1946-57 |
|  |  | Gambia 1965-93 |  |
|  |  | Georgia 2004- |  |
| Bavaria 1800-1817  Prussia 1800-05, 1813-39  Saxony 1806-12, 1815-30, 1845-47 | Baden 1841-47  Germany 1878-79 | Germany 1919-1932,  Germany West 1946-1990  Germany 1990- | Germany West 1949-90  Germany  1990- |
|  |  | Ghana 1979-80,  2001- |  |
|  |  | Greece 1864-1914,  1926-1935,  1944-48,  1975- | Greece 1880-1914,  1926-33,  1986- |
|  | United Province CA 1824-1838  Guatemala 1839-70, 1873-89,  1921-30 | Guatemala 1996- |  |
|  |  | Guinea-Bissau 2005- |  |
|  |  | Guyana 1992- |  |
| Haiti 1971-1985 |  | Haiti 1990, 1994-98, |  |
|  | United Province CA 1824-1838  Honduras 1839-51, 1854-1906,  1908-11, 1913-18, 1920-23, 1925- | Honduras 1982-84 |  |
|  | Hungary 1867-1917, 1920-43 | Hungary 1990- | Hungary 1990- |
|  |  | India 1950- |  |
|  | Indonesien 1950- | Indonesia 1999- |  |
| Iran 1800-1905, 1955-78 | Iran 1947- |  |  |
|  | Iraq 1924- |  |  |
|  |  | Ireland 1921- | Ireland 1927-32,  1952- |
|  |  | Israel 1948- | Israel 1948-66 |
| Modena 1815-60  Sardinia 1815-47  Tuscany 1815-47, 1849-60  Two Sicilies 1816-19, 1821-60 | Italy 1861-1921 | Italy 1947- | Italy 1948- |
|  |  | Jamaica 1959- | Jamaica 1959-92 |
| Japan 1800-57 | Japan 1868-1944 | Japan 1952- | Japan 1952- |
| Jordan 1946-50 | Jordan 1951- |  |  |
|  |  | Kenya 2002- |  |
|  | Korea 1800-1910 |  |  |
|  | Korea South 1948- | Korea South 1960,  1988- |  |
|  |  | Laos 1957-59 |  |
|  | Latvia 1929-33 | Latvia 1920-1933,  1991- |  |
|  | Lebanon 1943- | Lebanon 2005- |  |
|  |  | Lesotho 1966-69,  1993-97,  2001- |  |
|  |  | Liberia 2006- |  |
|  |  | Lithuania 1991- | Lithuania 1991- |
|  |  | Macedonia 1991- |  |
|  |  | Madagascar 1992- |  |
|  |  | Malawi 1994-2000,  2004- |  |
|  |  | Malaysia 1957-68 | Malaysia 1957-68 |
|  |  | Mali 1992- |  |
| Marocco 1800-1912, 1965-76 | Marocco 1800-1912 |  |  |
|  |  | Mauritius 1968- | Mauritius 1992- |
|  | Mexico 1822-33,  1835-45,  1848-61,  1864-75, 1917-29 | Mexico 1997- |  |
|  |  | Moldova 1993- |  |
|  |  | Mongolia 1992- | Mongolia 1996- |
|  |  | Mozambique 1994- |  |
|  |  | Myanmar (Burma) 1948-61 |  |
|  |  | Namibia 1990- |  |
| Nepal 1800-45, 1960-61 | Nepal 1800-1946 | Nepal 1999-2001,  2006- |  |
| Netherlands 1815-39 | Netherlands 1815-39 | Netherlands 1917-1939,  1945- | Netherlands 1917-39,  1945- |
|  |  | New Zealand 1857- | Nya Zealand 1857-75, 1893- |
|  | United Province CA 1824-1838  Nicaragua 1838-1925, 1928-35 | Nicaragua 1990- |  |
|  |  | Niger 1992-95, 2004- |  |
|  |  | Nigeria 1960-65,  1979-83 |  |
|  |  | Norway 1898-1939,  1945- | Norway 1898-39,  1945- |
| Oman 1800-1990 |  |  |  |
|  |  | Pakistan 1956-57,  1973-76,  1988-98 |  |
|  |  | Panama 1989- |  |
|  |  | Papua New Guinea 1975- | Papua New Guinea 1975- |
|  | Paraguay 1870-1939, 1947- | Paraguay 1992- |  |
| Parma 1815-60 |  |  |  |
|  | Peru 1821-23,  1828-80, 1883-1918, 1933-47 | Peru 1980-91,  2001 |  |
|  | Philippinerna 1944- | Philippines 1987- |  |
|  |  | Poland 1918-25,  1991- | Poland  2002- |
| Portugal 1800, 1802-06 | Portugal 1823-54, 1906, 1911-25 | Portugal 1911-25, | Portugal 1982- |
| Qatar 1971- |  |  |  |
| Russia 1800-1904 | Russia 1906-22 | Russia 1992,  2000-06 |  |
| Saudi Arabia 1926- |  |  |  |
|  |  | Senegal 2000-07 |  |
|  |  | Sierra Leone 1961-66,  2007 |  |
|  |  | Singapore 1959-62 |  |
|  |  | Slovak Republic 1993- | Sloval Republic 2006- |
|  |  | Slovenia 1991- | Slovenia 1991- |
|  |  | Solomon Islands 1978-99,  2004- |  |
|  |  | Somalia 1960- |  |
|  |  | South Africa 1992- |  |
| Spain 1800-07, 1814-1819, 1823-35 | Spain 1820-35,  1837-67, 1871-73, 1931-38 | Spain 1900-1922,  1930-38, | Spain  1982- |
|  |  | Sri Lanka 1948-81,  2001-02,  2006- |  |
|  |  | Sudan 1956-57,  1965-68,  1986-88 |  |
| Sweden 1800-1808 |  | Sweden 1914- | Sweden 1917 |
|  |  | Switzerland 1848- | Switzerland 1848- |
|  | Syria 1950 | Syria 1954-57 |  |
|  |  | Taiwan 1992- | Taiwan 2004- |
| Thailand 1800-1931 | Thailand 1935-40, 1942- | Thailand 1992- |  |
|  |  | Trinidad 1962-2005 | Trinidad 1997- |
| Turkey 1800-1907 | Turkey 1908-17 | Turkey 1946-53,  1960-70,  1973-79,  1983- |  |
|  |  | Uganda 1962-65 |  |
|  |  | Ukraine, 1991-92,  1994- |  |
|  |  | United Kingdom 1880- | United Kingdom 1922- |
|  |  | United States 1809- | United States 1845-49, 1871- |
|  | Uruguay 1830- | Uruguay 1952-70,  1985- | Uruguay  1999- |
|  | Gran Colombia 1821-30  Venezuela 1830-1908, 1941- | Venezuela 1958-2005 |  |
|  | Vietnam South 1955-64, 1973-75 |  |  |
|  | Yemen 1993- |  |  |
| Yemen North 1918-61 | Yemen North 1948-61 |  |  |
| Yugoslavia 1929-33 | Serbia 1838-57, 1861-66, 1869-1902 | Yugoslavia 2000-2002  Serbia and Montenegro 2003-06  Montenegro 2006-  Serbia 2006- |  |
|  | Zambia 1964-71 | Zambia 1991-95 |  |
|  | Zimbabwe 1980-82, 1999- |  |  |

Note: nation-states are indicated on the basis of the Polity IV data set. However, in cases where previously existing nation-states were parts geographically of later nation-states or when later nations-states are geographically parts of previous nation-states, the previous are listed in the same row as the later. Only those nation-states that have had all the core institutions of the three core dimensions are listed.
